# Experiences of family members caring for a sibling with mental illness in Giyani, Limpopo

**DOI:** 10.4102/curationis.v45i1.2347

**Published:** 2022-11-08

**Authors:** Tintswalo Vukeya, Annie Temane, Marie Poggenpoel

**Affiliations:** 1 Department of Nursing, Faculty of Health Sciences, University of Johannesburg, Doornfontein, Johannesburg, South Africa

**Keywords:** caring, experience, family member, mental illness, sibling

## Abstract

**Background:**

Mental illness is a serious condition affecting the diagnosed individual and all family members. Family members caring for a sibling with mental illness encounter severe challenges, which, if unresolved, becomes a burden they carry for the rest of their lives.

**Objectives:**

The purpose of the study was to describe family members’ experiences caring for a sibling with mental illness in Giyani.

**Method:**

A qualitative, exploratory, descriptive and contextual research design was used. Eight family members caring for a sibling with mental illness were purposively sampled. Data were collected through in-depth phenomenological interviews, and data were analysed using thematic coding, and an external coder was consulted.

**Results:**

The study’s findings revealed that family members caring for a sibling with mental illness experienced significant challenges. The three themes that emerged after data analysis were the following: caring for a sibling with mental illness was an overwhelming experience; family members experience emotional instability as a result of caring for their sibling with mental illness and family members need support in caring for their sibling with mental illness.

**Conclusion:**

The findings indicated that family members need support in caring for a sibling with mental illness. Family members would cope better with support from extended family, healthcare professionals in mental health and the community; this will reduce the burden of caring for a sibling with mental for family members. Furthermore, the availability of resources can assist in facilitating family members’ mental health.

**Contribution:**

This study could make an impact in psychiatric nursing practice, nursing education in promotion of support for family members for a sibling with mental illness.

## Introduction

This article focuses on family members’ experiences of caring for a sibling with mental illness in Giyani, Limpopo province. Mental illness may have a negative effect on family members, especially those who continuously interact with a sibling with mental illness. Families are the primary source of meaning in life and socialisation, but this role is disrupted when a sibling is diagnosed with mental illness. According to Uys and Middleton (2014:88), family is the main resource of the person diagnosed with a mental illness. In addition, caring for someone with a chronic condition is very demanding, but the burden becomes worse when caring for a sibling with mental illness because of fundamental disturbances in perception and thinking. Janardhan et al. (2015:185) highlighted that the onset of mental illness in any family is often, understandably, a time of turmoil, and most family members are ill-prepared to deal with mental illness in their families.

Mental illness is defined as maladaptive responses to stressors from the internal or external environment, evidenced by thoughts, feelings and behaviours that are incongruent with local and cultural norms, and that interferes with the individual’s social, occupational or physical functioning (Townsend 2015:16). In this article, mental illness refers to any psychiatric condition or disorder that causes significant distress and impairment in social, work or family activities; these conditions include and not limited to schizophrenia, bipolar mood disorder, anxiety disorder and depression.

According to a report published by News24 (Msomi 2022:np), mental health in South Africa is ranked among the worst in the world. This was aggravated by the Life Esidimeni tragedy in 2016 where chronic psychiatric patients were transferred from mental health institutions to nongovernment organisations with the aim to deinstitutionalise the chronic psychiatric patients; during the transfers 144 psychiatric patients died because of neglect and starvation (Section 27 2022:np).

The South African College of Applied Psychology (2018:np) indicates that one in six South Africans suffers from anxiety, depression or a substance use disorder, 40% of South Africans living with HIV have a comorbid mental disorder, 41% of pregnant women are depressed, about 60% of South Africans could be suffering from posttraumatic stress while only 27% of South Africans with severe mental disorders receive treatment; this is an indication of how mental illness has been neglected by the health system in South Africa.

Mental illness can be a disruptive and traumatic event for the diagnosed individual and negatively impact their family members. Because of changes or alterations in a sibling’s thoughts, cognition and behaviour, family members may feel stressed and overburdened when caring for their sibling with mental illness. According to Uys and Middleton (2014:89), continuous, long-term caregiving leads to significant stress that is often referred to as ‘family burden’ or ‘caregiving burden’. Moreover, sibling relationships can be a great source of meaning, enjoyment and mutual support, but they can also be challenging once someone finds out that their sibling has a mental illness. In such instances, they must learn to cope with this altered dynamic, which adds a new dimension to the sibling relationship (Griffiths & Sin 2013:808). Modise, Mokgaola and Sehularo (2021:7) found that one of the ways to cope is by the family members’ acceptance of the siblings’ condition, respect, effective communication with the sibling, prayer and counselling to relieve stress.

Family members play a significant role in caring for a sibling with mental illness, as people with mental illness are often discharged back home from the hospital. For many with mental illness, family is the only support system helping them to live at home and reintegrate into their community. A study conducted in the United States (Grossman & Magana 2016:238) revealed that family members are primary, and frequently unpaid, sources of support for people with disabilities, assisting with tasks that promote community living and integration across their life course. They also added that families help individuals lead meaningful lives in the community, including in terms of educational attainment and employment, and avoiding unnecessary and undesired institutionalisation.

Healthcare professionals often try to include families in caring for individuals with mental illness, but family inclusiveness focuses mainly on parents, overlooking the needs of overall family members, particularly siblings. Janardhana et al. (2015:185) also discovered that the experiences of families facing different types of mental illness had not been adequately studied, and their strengths had not been optimally utilised in assisting people with mental illness.

Sin et al. (2012:57) similarly reported that an understanding of siblings’ experiences and needs meant effective services could be developed. This view is supported by Park and Lee (2017:97), who found that limited information is available about sibling caregivers because existing studies focus on other family relationships, such as those with parents, spouses and children. Besides the study by Young and Flanagan (2021), there is a paucity of literature regarding the experiences of siblings caring for their sisters or brothers in South Africa. To fill this knowledge gap, the purpose of this study was to describe family members’ experiences caring for a sibling with mental illness in Giyani.

## Problem statement

The condition of mental health in South Africa is concerning and critical measures are needed. This lack of access to, as well as the quality of, mental health services is not unique to South Africa, but is a worldwide phenomenon and is found in almost all countries. This means according to Yogan (2019:463) that a global movement is urgently needed. It is important that changes are started and implemented in the communities. As stated in the introduction, only 27% of persons living with a mental illness receive treatment; this is not only a problem to the individual but also spreads to the family caring for the mentally ill.

Mental illness is a challenge that causes substantial problems in the family. It may have a negative impact on family relationships, finances, physical and psychological well-being of family members in terms of decreasing the quality of life of individuals caring for a sibling with mental illness. Modise et al. (2021:7) found that family members in their study experienced diverse challenges which included aggression from their mentally ill relatives, stigma from the community as well as negative attitudes from the nursing staff.

The researcher, who was working in an acute psychiatric ward at a public hospital at the time of data collection, had interacted with many family members accompanying their sibling with mental illness on admission when they were coming to visit them at the hospital, and also when conducting home visits around Giyani. From these encounters, the researcher was able to identify that family members caring for a sibling with mental illness were experiencing several challenges. Some family members would burst into tears during conversations as a result of their situation. Based on the overwhelming challenges these family members experienced, the researcher wanted to explore the phenomenon in greater depth.

## Research purpose

The study’s purpose was to describe family members’ experiences caring for a sibling with mental illness in Giyani.

## Research design and method

According to Polit and Beck (2020:743), the research design is the overall plan for addressing a research question, including specifications for enhancing the study’s integrity. To explore, describe and understand family members’ experiences caring for a sibling with mental illness, a qualitative design (which is exploratory, descriptive and contextual) was used, embracing a phenomenological approach.

### Setting

The researcher collected data in the field at the site where participants experienced the problem under study, which was considered the natural setting (Creswell & Creswell 2018:181). Five interviews were conducted telephonically, and three were conducted at participants’ homes in Giyani, Limpopo province, South Africa. The participants were rich informants of the research context as they were the ones taking care of their siblings on a daily basis. The up-close information gathered by talking directly to people and seeing them behave and act within their context enhanced the researcher’s understanding of the family members’ experiences (Creswell & Poth 2016:37).

### Population and sample

The research population is defined as an entire set of individuals or objects with some common characteristics (Polit & Beck 2020:139). This study’s population was family members caring for a sibling with mental illness. A purposive sampling method, as described by Gray, Grove and Sutherland (2017:34), was used, and the researcher consciously selected specific participants to include in the study. A sample was built to gain a deeper understanding of family members’ experiences caring for a sibling with a mental illness. The researcher who worked at the psychiatric ward requested assistance from the unit manager of the psychiatric ward to recruit willing participants for the study; the researcher had no relationship with the participants. The inclusion criteria were family members assisting and supervising a sibling with a mental illness, living or not living in the same house. Participants were 18 years and older, had access to a cellphone or landline, and were able to communicate in English or Xitsonga. The exclusion criteria were family members who were not caring for siblings with a mental illness and also those who were not willing to participate in the study. These exclusion criterion made participants ineligible to partake in the study.

### Data collection

Data were collected by identifying subjects through the precise, systematic gathering of information relevant to the research purpose (Grove, Gray & Burns 2015:502). In-depth phenomenological interviews were ultimately conducted with eight family members between November 2020 and April 2021. In addition, field notes were taken by the researcher to collect data. These field notes consisted of personal, methodological, observational and theoretical notes (Gray et al. 2017:256–257). The main question posed to participants was: ‘What is it like to care for a sibling with mental illness?’

The researcher gave participants ample time to express their views and experiences, and the interviews lasted 40–60 min. The interviews were conducted in the interviewees’ preferred home language in Xitsonga and were audio-recorded and transcribed by the researcher.

### Data analysis

The purpose of qualitative data analysis is to organise, provide structure to and elicit meaning from data, as highlighted by Holloway and Galvin (2017:287). Data analysis was done in the original language of the interviewees in Xitsonga, and the findings were translated into English by the researcher and the independent coder during the process of consensus discussion.

Data were also analysed using Tesch’s thematic coding method, comprising of eight steps (Creswell & Creswell 2018:196). The researcher read all transcriptions, field notes and observations to get a sense of the data, and then notes were made of the information. The researcher grouped similar topics to get an overall picture. Topics were abbreviated and placed into codes next to relevant interviews; related topics were grouped to reduce the total list of categories. The researcher assembled data belonging to each category in one place to perform a preliminary analysis and interpret the meaning of themes. The data were also given to a qualified and experienced independent coder, and the researcher met with the independent coder for a consensus discussion on the data analysis findings. A literature review then took place to support the meaning of themes and categories.

### Ethical considerations

Ethical approval to conduct the study was obtained from the Faculty of Health Sciences Research Ethics Committee, University of Johannesburg (reference number: REC-705-2020). Permission to conduct this research was also granted by the Limpopo Department of Health (reference number: LP202010-003) and Evuxakeni hospital. The ethical principles of autonomy, beneficence, nonmaleficence and justice (Dhai & McQuoid-Mason 2011:14–15) were adhered to throughout the study.

All participants were treated as autonomous agents with the freedom to conduct their lives as they chose without external control (Gray et al. 2017:162). The researcher made sure that participants were allowed to make free, independent choices to participate in or withdraw from the study if they wished (Rebar & Gersch 2015:136).

Informed consent was also obtained from participants before the interviews commenced. The researcher ensured that participants understood the contents of the informed consent form so they could make an informed decision. The researcher read the information letter for the participants and allowed them to ask questions about their participation in the study. Participants understood their participation was voluntary and that they could withdraw (without any penalties) if they felt uncomfortable continuing. The researcher ensured that the research was not intrusive, and participants’ identities were kept anonymous, and data collected were kept private to ensure privacy and confidentiality. Ultimately, the collected data were only accessible to the researcher, the two supervisors and the independent coder. The independent coder signed a confidentiality agreement before commencing with data analysis.

According to Moule, Aveyard and Goodman (2017:102), non-maleficence is the principle of ‘doing no harm’ and preventing physical, psychological, emotional, social and economic harm. The researcher ensured that participants were protected against any harm to their mental health, psychological well-being, personal values and dignity. In addition, the researcher indicated to the participants that if at any time during the interviews they experience emotional distress they will be referred for counselling at a public mental hospital. The five face-to-face interviews were conducted at the homes of the participants, and physical harm was prevented by applying coronavirus disease 2019 (COVID-19) protocols such as 1.5-meter distancing to prevent COVID-19 infections. Furthermore, participants were also not exposed to any form of exploitation, and no information was divulged against their will or used against them. Moreover, researchers have a duty to minimise harm and maximise benefits to participants. The researcher ensured that the benefits of participating in the study outweighed the risks for participants, as described by Polit and Beck (2020:139).

### Measures to ensure trustworthiness

Measures to ensure trustworthiness – credibility, transferability, dependability and confirmability – were adhered to throughout the study (Lincoln & Guba 1985:301). To ensure credibility, the researcher employed prolonged engagement with participants, persistent observation, peer debriefing and member checking (Houser 2012:425; Lincoln & Guba 1985:304). Lincoln and Guba (1985:316) refer to ‘transferability’ instead of ‘generalisation’, which means that the findings in one context can be transferred to a similar situation or similar participants (Holloway & Galvin 2017:309). This study presented a description of the findings, with supporting direct quotations from participants. The participants’ demographics were also densely described.

The term ‘dependability’, instead of ‘reliability’, was used by Lincoln and Guba (1985:289). It means research findings should be consistent and accurate to establish the study’s trustworthiness. For the findings to be dependable, they should be consistent and accurate (Holloway & Galvin 2017:309). In this study, the findings’ accuracy was ensured, an audit was done to examine the process, and a dense description of the research methodology was provided.

To attain confirmability, there was an internal agreement between the researcher’s interpretation and actual evidence. In addition, an audit was done of the entire research process to ensure the findings reflected participants’ voices and not the researcher’s perspectives.

## Findings

### Description of participants

Eight family members caring for a sibling with mental illness in Giyani were interviewed. Because of COVID-19 restrictions, five interviews were conducted telephonically in order to promote safety and three interviews were face-to-face as per the participants’ preference. Eight participants took a parental role in caring for their sibling because their parents had passed away or were too old to care for their child with mental illness. A majority of participants had a low-income status; they were unemployed and dependent on their sibling’s disability grant or their parents’ social grant. The participants’ demographics are presented in Table 1.

**TABLE 1:** Participants’ demographics.

Participant	Gender	Age	Years in caring	Marital status	Relationship to the sibling with mental illness
Participant 1	Male	36	18	Married	Brother
Participant 2	Female	55	25	Divorced	Sister
Participant 3	Female	46	16	Married	Sister
Participant 4	Male	50	24	Married	Brother
Participant 5	Female	62	16	Married	Sister
Participant 6	Male	23	6	Single	Brother
Participant 7	Female	49	12	Married	Sister
Participant 8	Female	28	8	Single	Sister

*Source:* Vukeya, T.C., Temane, A. & Poggenpoel M. 2022, ‘Experiences of family members caring for a sibling with mental illness’, Master’s dissertation, University of Johannesburg, p. 48

The findings were based on the themes presented in Table 2.

**TABLE 2:** Themes of family members’ experiences caring for a sibling with mental illness.

Themes	Experiences
Theme 1	Family members find caring for a sibling with mental illness to be overwhelming.
Theme 2	Family members experience emotional instability as a result of caring for their sibling with mental illness.
Theme 3	Family members need support in caring for their sibling with mental illness.

*Source:* Vukeya, T.C., Temane, A. & Poggenpoel M. 2022, ‘Experiences of family members caring for a sibling with mental illness’, Master’s dissertation, University of Johannesburg, p. 48

### Theme 1: Family members find caring for a sibling with mental illness to be overwhelming

Participants felt overwhelmed as a result of the factors explained in the subthemes.

#### Subtheme 1.1: Unpredictability of the mental illness

Family members expressed caring as an overwhelming experience because of the unpredictable nature of mental illness, their siblings’ relapses because of noncompliance, and aggressive outbursts. Family members said that caring for a sibling with mental illness is very challenging and affects the way they live and behave. Some said they required chronic treatment because of stress or were divorced from their spouse because of their sibling’s behaviour. The unpredictability of their sibling’s behaviour left them frustrated and overwhelmed, not knowing what to expect, making them fearful. They said:

‘It is very much hard when that situation comes you can be traumatized. It was hard to cope with the kind of situation we facing it is really hard.’ (Participant 6, 23 years, male, 6 years of caring, single, brother)

‘Eish … it is very difficult because every morning you have to check that how he is, so that you know how to respond according to his mood and behaviour … life is very difficult and painful.’ (Participant 2, 55 years, female, 16 years of caring, divorced, sister)

Such overwhelming experiences could potentially affect how they live and behave. One family member was worried about her children’s safety when they were around her sibling with mental illness:

‘His behaviour can change anytime I don’t know what can happen to my children if this thing happens in my absence.’ (Participant 7, 49 years, female 12 years of caring, married, sister)

Other family members also shared how their sibling’s unpredictable behaviour affects them and the community in which they reside. Some said their sibling is a danger at home and in the community. Family members mentioned their sibling sometimes starts chasing and assaulting people for no reason; others said their sibling randomly picks up objects and throws these at people without being provoked. The following are some of the statements family members shared:

‘It is very much difficult sometimes he starts chasing people in the community he even assaults innocent young children for nothing, he is very troublesome.’ (Participant 4, 50 years, male, 24 years of caring, married, brother)

‘Sometimes you will just be sitting then unexpectedly take a stone and throw it at you.’ (Participant 1, 36 years, male, 18 years of caring, married, brother)

#### Subtheme 1.2: Relapse because of noncompliance

Family members indicated that another problem that caused them to feel overwhelmed was their sibling’s noncompliance. Participants said their sibling was noncompliant with treatment at home, and it was frustrating because they knew compliance promoted their sibling’s appropriate behaviours. One participant said it was hard to force her sibling to comply with treatment because he became defensive and aggressive:

‘He didn’t want to take his treatment … and started not coming home at night, he will be walking throughout the night talking to self sometimes goes to my sisters’ house and come to my house talking.’ (Participant 3, 46 years, female, 16 years of caring, married, sister)

Another stated:

‘It is very difficult when he relapses no one is able to help him … he doesn’t take his treatment.’ (Participant 4, 50 years, female, 24 years of caring, married, brother)

#### Subtheme 1.3: Siblings displayed aggressive outbursts

Aggressive outbursts were another challenge family members experienced. They constantly lived in fear of their sibling’s aggressive behaviour, often with unknown causes. Some said their siblings were very demanding, and they sometimes demanded impossible things. A few participants mentioned their siblings asked for money and became aggressive if their request was denied. Other family members said their sibling became aggressive towards them for no apparent reason:

‘We do not understand his behaviour; sometimes he just threatens us that he will beat us up for nothing.’ (Participant 8, 28 years, female, 8 years of caring, single, sister)

‘When she tries to talk to him he just grabs anything he finds and hit her with.’ (Participant 3, 46 years, female, 16 years of caring, married, sister)

One participant said when his brother was angry, he became destructive and broke things at home or the house itself. He explained that he became frustrated when his brother was aggressive because there was nothing they could do; they just watched him do whatever he wanted:

‘He broke all the windows in the house, also pulling out window frames it was a mess in the house we didn’t know what to do and he is tough guy we wouldn’t fight or even stop what he was doing, we had to leave him to do whatever that that he wanted to do in the house destroying things.’ (Participant 6, 23 years, male, 6 years of caring, single, brother)

### Theme 2: Family members experience emotional instability as a result of caring for their sibling with mental illness

Caring for a sibling with mental illness was stressful for family members, and emotional instability was another major concern participants mentioned during interviews; their experiences were emotional. Family disequilibrium also extended to community members, and participants spoke about financial instability related to the cost of caring for their sibling with mental illness.

#### Subtheme 2.1: Fear related to the sibling’s behaviour, hopelessness, helplessness and concern over the sibling’s safety

Family members caring for a sibling with mental illness were constantly worried about community members’ safety as their sibling became aggressive and violent at times. This behaviour was disturbing to family and community members. Participants said they lived in fear when their sibling was at home, thinking they could hurt those around them (even younger children) for no apparent reason. Family members stated that:

‘There is no one who is not afraid of him, immediately when he comes home even those people who usually comes home would not come because of him, and he knows it very well that people are afraid of him.’ (Participant 2, 55 years, female, 18 years of caring, divorced, sister)

‘To tell you the truth they are afraid of him … Even my own kids are afraid of him because they have seen everything he does when he relapses destroying things here at home.’ (Participant 4, 50 years, male, 24 years of caring, married, brother)

Concern over their sibling’s safety was also reported by family members since those diagnosed with a mental illness are more likely to be abused in the community because of their mental state. Such abuse ranges from physical, sexual and psychological abuse. During the interviews, one family member said her brother was used by his friends, who forced him to break into people’s houses. Then, once he was caught, he was assaulted.

Family members shared some experiences related to their constant worry about their sibling’s safety by stating:

‘The thought of not knowing what might be happening to him, or maybe he is killed already is very painful.’ (Participant 1, 36 years, male, 18 years of caring, married, brother)

‘We always scared we don’t know what will happen to him, the community can hurt or kill him, community members will hurt him or even kill him if he comes home.’ (Participant 4, 50 years, male, 24 years of caring, married, brother)

Concern over a sibling’s safety caused many to experience anxiety, especially when siblings wandered from home and became disruptive and violent.

#### Subtheme 2.2: Family disequilibrium extending to community members

Family members experienced instability within the family and community because of their sibling’s behaviour; the phenomenon often resulted from relapse. Participants mentioned that other siblings would chase them from their own home, and they would be forced to seek safety from their neighbours:

‘So we were supposed to move out of the house and leave him alone, so it is very difficult at times.’ (Participant 8, 28 years, female, 8 years of caring, single, sister)

Caring for a sibling with mental illness was reported as challenging and burdensome, worsened by a lack of support from family members. Some participants remarked:

‘There is no support you have to deal with the situation by yourself when you contact relatives everyone would say they are not home while others say they are afraid of him.’ (Participant 6, 23 years, male, 6 years of caring, single, brother)

‘He is very dangerous and there is no support where I stay, they just watch and do not do anything even if … there is no support in my community you just face the situation alone you are on your own.’ (Participant 3, 46 years, female, 16 years of caring, married, sister)

In addition to the challenges family members faced while caring for a sibling with mental illness, which interfered with their day-to-day work, they also dealt with societal challenges. Participants felt they were not treated like other people within their community. They complained they were discriminated against and stigmatised in their community, which resulted in psychological distress and low quality of life:

‘Community members hate us, and when the king can say they no longer want us there it means we suppose to relocate because he is troublesome when he is home.’ (Participant 4, 50 years, male, 24 years of caring, married, brother)

‘It is true people hate us when someone is mentally ill … those people whom he destroyed their properties will not be happy because it’s true he destroyed their things but we pay back and fix whatever he destroyed.’ (Participant 5, 62 years, female, 16 years of caring, married, sister)

#### Subtheme 2.3: Financial instability related to the cost of caring for a sibling with mental illness

Family members were subjected to stress and frustration caused by the demanding nature of caring for a sibling with mental illness. They ensured their sibling’s treatment and follow-ups, but the most frustrating challenge was their sibling’s behaviour over finances. The continuous demands they made were unbearable, ranging from demanding money by force to buy substances, misusing their own disability grants and forcing family members to take out loans from loan sharks. Participants further reported that reimbursements for damages their sibling might have caused during relapse were significant. Participants shared:

‘It became extremely difficult when he comes home because we had to pay for his medical expenses and also taking him to pastors whom some of them demanded to be paid before they prayed for him.’ (Participant 1, 36 years, male, 18 years of caring, married, brother)

‘Sometimes he will demand the last money reserved for bread or something valuable in the house but you will be bound to give him.’ (Participant 2, 55 years, female, 18 years of caring, divorced, sister)

For some, financial strain arose as a result of their sibling selling the family’s household appliances:

‘Most of the money is spent on him as he has a tendency of losing or selling his phone and also take our cell phones sell them so to be honest is difficult to care for him.’ (Participant 8, 28 years, female, 8 years of caring, single, sister)

One family member elaborated on how his younger brother treats his mother, who is a pensioner:

‘He wakes up at 04H00 in the morning every day and tells her to go to loan sharks to borrow money for him to buy alcohol and cigarettes, and indeed she will go and ask for the money he is demanding.’ (Participant 1, 36 years, male, 18 years of caring, married, brother)

Another financial implication was having to replace damaged items resulting from siblings’ aggressive outbursts. Participants explained they struggled to replace damaged goods:

‘Previously he destroyed other people’s properties though things he destroyed did not cost too much that is why we managed to repay I don’t have money to replace everything.’ (Participant 6, 23 years, male, 6 years of caring, single, brother)

‘Sometimes we find ourselves not having enough money to replace damages caused and also to pay transport to locate and bring him home.’ (Participant, 1, 36 years, male, 18 years of caring, married, brother)

### Theme 3: Family members need support in caring for their sibling with mental illness

Participants encountered a lack of support from family members and health facilities, leaving them feeling hopeless at times. In addition, most participants wanted the government to assist them. Family members also expressed the need for home visits and more information about mental illness, including a hotline for emergency support and places of safety.

#### Subtheme 3.1: Need for home visits

Home visits by health professionals emerged as a major need for most family members caring for a sibling with mental illness. These visits would be conducive to monitoring the sibling, checking how family members are coping with them at home, and providing support and reassurance to those caring for a sibling with mental illness. Family members believed their sibling’s behaviour could be modified if they knew healthcare practitioners might unexpectedly visit:

‘We also asking for social workers and nurses to come visit us sometimes.’ (Participant 1, 36 years, male, 18 years of caring, married, brother)

‘I think health professionals must come visit us regularly, because most of the times when he destroys people’s properties it always becomes our responsibility.’ (Participant 4, 50 years, male, 24 years of caring, married, brother)

#### Subtheme 3.2: Need for more information about mental illness, including a hotline for emergency support

Participants mentioned the need for more information about chronic mental illness, including a hotline for emergency support. Some raised the issue of insufficient or a lack of information as a very serious and contributory factor to them being stigmatised within the community. Family members claimed that the ill-treatment they received within their communities might be because of a general lack of information and understanding about mental illness:

‘If maybe there was psychoeducation about mental illness in our community maybe there will be an understanding and even us we will know what to do when we face these challenges.’ (Participant 1, 36 years, male, 18 years of caring, married, brother)

A family member claimed that an emergency hotline would be helpful. Individuals could call the hotline and get immediate and urgent assistance without being judged:

‘If there was an emergency number to call when he becomes aggressive so that they come and take him it will be much better.’ (Participant 4, 50 years, male, 24 years of caring, married, brother)

Another elaborated:

‘Maybe also explaining this mental illness and the signs and how are we supposed to help or do when we encounter such situation.’ (Participant 6, 23 years, male, 6 years of caring, single, brother)

#### Subtheme 3.3: Need for a place of safety for the sibling with mental illness

A place of safety was one of the needs family members raised because they claimed their siblings were difficult to manage at home after being discharged. Family members stated that a place of safety within their communities (where they can visit their sibling regularly) could assist them in caring for their sibling with mental illness. They shared:

‘If there can be place of safety to keep them and we only go there to check on them.’ (Participant 5, 62 years, female, 16 years of caring, married, sister)

‘If the government can have a place to keep this people so that we only go there to check on them as relatives.’ (Participant 3, 46 years, female, 16 years of caring, married, sister)

Some also said it is a relief when their sibling is hospitalised, knowing they are safe. Siblings with mental illness sometimes leave home for days without telling anyone, causing family members to have sleepless nights.

## Discussion

The study’s objective was to explore and describe family members’ experiences caring for a sibling with mental illness. In this study, participants shared that it was an overwhelming experience because of the unpredictability of mental illness. The situation was exacerbated by a lack of understanding of mental illness, relapses from noncompliance, aggressive outbursts from the sibling, financial disruption as well as social exclusion from their communities.

Participants in this study find caring for a sibling with mental illness to be overwhelming. They expressed their sibling’s behaviour would change from time to time; this left them frustrated, not knowing what to expect. Von Kardoff et al. (2016:4) identified that most of their participants acknowledged they experienced uncertainty because of the unpredictable nature of and ambivalence about mental illness and the treatment process. Affected individuals’ unpredictable behaviour made it difficult for family members to cope. This view was supported by Iseselo, Kajula and Yayha-Malima (2016:6), who claim family members expressed concern that there was no one else able to handle this unpredictable behaviour.

Participants in this study reported that their siblings became aggressive when discussing treatment, and they said it was difficult because they could not force their compliance. In a study by Pusey-Murray and Miller (2013:119), family members reported that mentally ill patients sometimes refused to take their medication as prescribed; unpleasant side effects and a lack of insight were the primary reasons for this. This is similar to a South African study by Modise et al. (2021:5–6) who mentioned defaulting on medication as challenge family members of psychiatric patients were facing which led to aggression, unpredictable behaviour, stigma and discrimination by the community. Moreover, this was also reported by Von Kardoff et al. (2016:4), stating medication compliance was one of the problems reported by their participants.

A South African study by Monyaloue et al. (2014:12) found that family members caring for a sibling with mental illness were concerned with some of their sibling’s behaviours. These concerns are related to verbal abuse, damage to property, poor personal hygiene and disrupting other people’s lives. Family members were understandably tormented by the threat of aggressive acts. Gunderson (2011:8) found that participants in their study had reactions which varied from wanting to protect the patient, to anger at the perceived attention-demanding aspects of the behaviour. During interviews, it became clear that families felt at risk of being injured or killed by their violent and aggressive mentally ill family members, and as such, families lived in fear (Monyaloue et al. 2014:5).

Family members in this study expressed emotional instability as a result of caring for their sibling with mental illness. The family members experienced emotional instability resulting from the impact of caring for a sibling with mental illness. This emotional instability was attributed to participants’ concern over their siblings’ safety. Moreover, instability extended to community members, caused by a lack of support from family members, poor relationships and engagement with community members. Emotional instability was also related to the financial costs of caring for their sibling. Family members in this study expressed that the continuous demands on the finances were unbearable. According to a study conducted by Ae-Ngibise et al. (2015:5), family members who are unable to work often depend on distant relatives for financial support. Some family members expressed hopelessness in making economic gains while caring for their sibling with mental illness. Chang et al. (2016:4) also reported that being unemployed had a significant impact on caregivers’ finances.

Family members’ concern for the safety of their mentally ill siblings caused many to experience anxiety, especially when their sibling went missing or became disruptive and violent. According to Bowman et al. (2017:6), adults’ reports of less warmth in their relationship with their ill siblings were associated with negative overall caregiving experiences. In support, Farrel and Krahn (2014:8) stated that from a life course perspective, the severity of a loved one’s mental illness could shape the experience of all family members in fundamental ways.

Participants in this study expressed a need for support in caring for their sibling with mental illness. This is supported by Von Kardoff et al. (2016:3) who stated that the most significant problems experienced by family members are the lack of help and support from family and acquaintances, lack of understanding, empathy and conflict, and lack of being supported in the caregiving role by others. They were forced to bear the burden of caring for a patient with severe mental illness, along with undertaking other responsibilities and tasks because of a lack of support resources (Ebrahimi et al. 2018:14). Ntsayagae, Myburgh and Poggenpoel (2019:5) similarly discovered that caregivers’ perspectives were mediated by an emotional sequel, feelings of being overburdened and their unmet support needs from family and healthcare professionals.

Participants in this study raised issues of insufficient or a lack of information which contributed to them being stigmatised within the community. Ebrahimi et al. (2018:15) concur with this finding as the researchers also discovered that the main reason family members did not disclose their sibling’s illness and isolated themselves from public view was their fear of being judged negatively by others. Moreover, participants in a study by Stutterheim and Ratcliffe (2021:8) reported experiencing social distancing and having fewer social networks because they had a sibling with mental illness. In addition, Greenwood, Mezey and Smith (2018:41) confirm that social exclusion has a negative impact on individuals’ quality of life. If unaddressed, stigma will continue to pose a major barrier to improving mental health (Rasmussen et al. 2019:3).

Participants in the current study expressed a need for support in caring for their sibling with mental illness. The participants expressed a need for a place of safety for their sibling as the community was not safe, a need for information as the community and themselves lack information as well as a need for home visits by healthcare professionals. This is supported by a study by Sin et al. (2015:1) who claim that psychoeducation will increase family members’ knowledge about mental illness. It will help them cope more effectively when providing care for their sibling with mental illness, and enhance their own well-being. Von Kardorff et al. (2016:2) stated that most of their participants did not have enough information about the disease or treatment process. The authors (Von Kardoff et al. 2016:4) also revealed that some participants were worried about the lack of government support and services.

Participants in the current study expressed the need for support in caring for their sibling with mental illness. Support was required in the form of home visits, information about mental illness, a hotline for emergency support and a place of safety for siblings with mental illness. Bowman et al. (2014:17) revealed that a home visit provides a valuable opportunity to build therapeutic relationships. It may indicate the healthcare team’s commitment to focus on the patient as an individual and take the extra effort to build a sound, meaningful relationship. In support, Chang and Chou (2015:7) stated that home visits provide a model for continuous treatment, lowering the rehospitalisation rate, average days of hospitalisation and healthcare costs.

### Recommendations

It is recommended that family members’ mental health is facilitated in caring for siblings with mental illness as early as possible. Ideally, this should happen as soon as the sibling is diagnosed because family members can be frustrated and shocked by the diagnosis. It is recommended for health professionals in mental health to provide psychoeducation about mental illness tailored to these family members caring for a sibling with mental illness. The psychoeducation may include aspects such as what is mental illness, causes thereof, signs and symptoms, signs of relapse and management as these can maybe ease the burden and promote the family members’ mental health.

Another recommendation is for healthcare professionals to conduct individual therapy, family therapy and group therapy for family members’ caring for a family member with a mental illness. Community awareness about mental illness should also be promoted, and financial assistance should be facilitated by establishing income-generating community projects.

Healthcare professionals could mobilise resources that can facilitate family members’ mental health by making resources available to them. Healthcare providers’ follow-up visits when a sibling is discharged, halfway houses and sheltered employment, as well as liaisons between the South African Police Service and hospitals, could assist family members in difficult times.

### Limitations

The first limitation of this study is that the findings were collected from a psychiatric ward in one of the public hospitals in a rural community. Thus, the findings from this study cannot be generalised to other hospitals or provinces. The second limitation that the researchers encountered was family members’ concerns about the interviews being audio-recorded; this would mean the researchers may have not captured all the important information required for this study.

## Conclusion

The findings in this study reveal that family members caring for a sibling with mental illness in Giyani have an overwhelming experience, experience emotional instability and expressed a need for support. Family members would cope better with support from extended family, healthcare professionals in mental health and the community. Thus, the burden of caring for a sibling with mental illness might be reduced for family members. The availability of resources like a place of safety for people with mental illness, halfway houses as well as sheltered employment can assist in facilitating family members’ mental health.
